# To approach or to avoid: The quadripolar model of achievement motivation revisited in a Confucian-heritage context

**DOI:** 10.3389/fpsyg.2022.1046775

**Published:** 2023-01-20

**Authors:** Guan Ying Li, Bih-Jen Fwu, Tong-Rong Yang, Yi-Kai Chen

**Affiliations:** ^1^Center for Teaching and Learning Development & Center for General Education, National Taiwan University, Taipei, Taiwan; ^2^Center for Teaching and Learning Development & Center for Teacher Education, National Taiwan University, Taipei, Taiwan; ^3^Department of Psychology, National Taiwan University, Taipei, Taiwan

**Keywords:** fear of failure, hope of success, academic risk-taking, emotional distress, indebtedness

## Abstract

Academic challenges and failure are inevitable in pursuit of higher education. According to the self-worth theory, trying hard but failing implies low ability that would be a threat to personal worth, thus preventing students from approaching academic challenges. Nevertheless, previous studies have shown that students in the Confucian-heritage contexts (CHCs) tend to persist rather than quit in the face of academic failure. According to the role obligation theory of self-cultivation (ROT), the CHC learners would perceive academic failure from personal and interpersonal perspectives. The former refers to personal obligations to exert oneself toward the ultimate good, and the latter refers to fulfilling filial obligations to parents by achieving academic excellence. Given the fundamental differences in learners’ perceptions of academic failure between the CHCs and the Western, Educated, Industrialized, Rich, and Democratic (WEIRD) contexts, this study examined the applicability of the quadripolar model of achievement motivation based on the self-worth theory in a CHC higher education institution. Results of confirmatory factor analysis (CFA) supported a two-factor model of fear of failure, including a personal and an interpersonal subfactor. Latent class analysis (LCA) showed that apart from the four existing categories of the quadripolar model, two additional CHC categories emerged and constituted half of the sample. The two CHC categories demonstrated different learner characteristics compared with their corresponding quadripolar categories in terms of levels of emotional distress and academic risk-taking tendency. The results may help debunk the myth that learner characteristics in the CHCs are identical to those observed in the WEIRD contexts. The fundamental differences in fear of failure further indicated the inadequacy of the self-worth theory in explaining achievement motivation in the CHCs where relationalism and role obligations are significant parts of the cultural traditions.

## 1. Introduction

Entering higher education is a milestone in the transition to adulthood. However, it also means that students will be confronted with greater academic challenges involving a higher degree of risk taking wherein failure and success might occur one after another. Although failure is inevitable in challenging academic environments, according to the self-worth theory of achievement motivation (hereafter the self-worth theory; Beery, 1975; Covington and Omelich, 1979; Covington, 1984, 1992), academic failures would be a threat to personal worth. Trying hard but failing implies low ability that would trigger humiliation and shame of students who regard incompetence as a fixed trait (Covington, 1984). This fixed mindset of ability would render students vulnerable to emotional distress and helplessness and would lead them to an avoidance tendency toward future academic challenges (Clifford, 1991; Covington, 1992; Martin and Marsh, 2003; De Castella et al., 2013).

Nevertheless, previous studies have shown that East Asian students tend to hold an incremental/growth mindset (Heine et al., 2001) and that students tend to persist rather than quit in the face of academic failure in the Confucian-heritage contexts (CHCs) (Fwu et al., 2018; Chen et al., 2021; Fwu et al., 2021). Given the cultural differences in responses to academic failure, it is reasonable to argue that the self-worth theory that was primarily based on the Western, Educated, Industrialized, Rich, and Democratic (WEIRD) samples might not be fully applicable to the CHCs (Henrich et al., 2010), where role obligations are central to achievement motivation of learners (Chen et al., 2009).

This study aims to examine the applicability of a quadripolar model of achievement motivation proposed by Covington and Omelich (1991) in a CHC context. The quadripolar model was grounded in the self-worth theory to categorize learners based on their responses to academic failure and success orientations. A re-examination of the quadripolar model in a CHC higher education institution would contribute to a better understanding of how CHC students respond to academic failure and challenges, both of which are inevitable in pursuit of higher education. The study would also uncover differences in learner characteristics between the CHCs and the WEIRD societies. We believe that only if cultural differences are considered, can insightful educational implications be made.

### 1.1. The quadripolar model of achievement motivation

**Figure 1:**
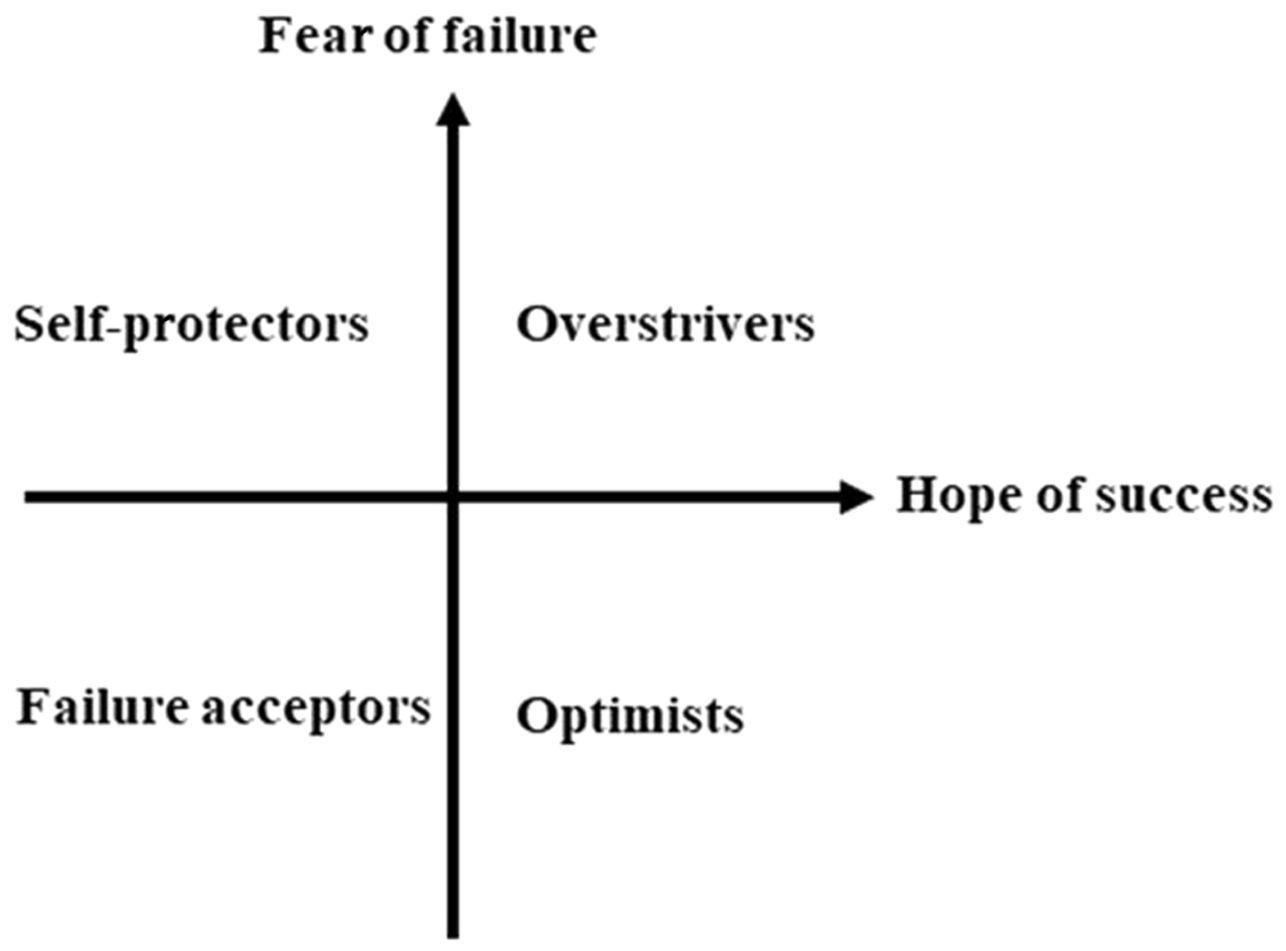
The quadripolar model of achievement motivation. Copyright © 2013 by American Psychological Association. Adapted with permission from De Castella et al. (2013).

Based on the self-worth theory, Covington and Omelich (1991) proposed a quadripolar model of achievement motivation based on the dynamics between fear of failure (FF) and hope of success (HS). Previous studies had used a bipolar model to differentiate between failure-fearing and success-oriented students by placing FF and HS on two extremes (Moulton, 1965; Clifford et al., 1988). The quadripolar model, on the other hand, was underpinned by the assumptions that FF and HS are two opposing but not mutually exclusive forces and that the two forces work in combination rather than in isolation. The dynamics between FF and HS have generated four learner categories: (1) overstrivers (high in both FF and HS), (2) self-protectors (high in FF, but low in HS), (3) failure acceptors (low in both FF and HS), and (4) optimists (low in FF, but high in HS; Covington, 1992), as shown in Figure 1.

The four categories shared some characteristics but differed to some extent (Covington and Omelich, 1991).The optimists and overstrivers both demonstrated a high level of success orientation. These students had an approach tendency toward learning, generally perceived themselves as competent, and were academically superior. However, overstrivers were also high in fear of failure. Their fears of appearing incompetent in the eyes of others would result in an avoidance tendency toward academic failure. Because the overstrivers were caught in the conflict between FF and HS, they were vulnerable to self-doubts and emotional distress. High fear of failure was also the characteristic of the self-protectors (Covington, 1992). These students had a strong avoidance tendency toward failure and suffered from anxiety due to their high fear of failure (Martin and Marsh, 2003). Failure acceptors represented “a blend of approach/avoidance tendencies” (Covington and Omelich, 1991, p. 103). These students were neither the most failure-fearing nor a success-oriented group, with their perceived ability and anxiety levels falling in between the overstrivers and self-protectors. Instead of interpreting these characteristics as indifference, Covington and Omelich (1991) suggested that the failure acceptors might turn to other sources for intellectual and emotional satisfaction than academic achievement.

The quadripolar model was examined mainly in the WEIRD contexts (Covington and Omelich, 1991; Covington, 1992; Martin and Marsh, 2003), with De Castella et al. (2013) as an exception. De Castella et al. (2013) examined the quadripolar model in a cross-cultural study, with the four categories and corresponding learner characteristics consistently emerging from the Japanese and Australian samples. De Castella et al. (2013) found that the self-protectors were at greatest risk among the four categories as measured by self-handicapping, defensive pessimism, learned helplessness, and disengagement from school. Self-protectors were prone to self-handicapping to deflect the cause of academic failure away from a lack of ability so as to protect their self-worth. Moreover, they felt the most helpless regarding their control over academic outcomes and thus being the most vulnerable to underachievement and disengagement from school. These results were consistent with those found in Covington and Omelich (1991). Although De Castella et al. (2013) noted that the Japanese and Australian samples represented “two highly distinct cultural settings” (p. 876), they did not explain clearly why consistent results were found across the two culturally distinct samples. Instead, the study concluded that “when it comes to protecting one’s self-worth, Eastern and Western students may have much in common” (De Castella et al., 2013, p. 874).

Despite that no cultural differences were observed in De Castella et al. (2013), previous studies have indicated that East Asian students had a stronger approach tendency toward challenges, compared with the Northern American students (Heine et al., 2001; Zhang and Cross, 2011). The consistent results found in De Castella et al. (2013) might be a result of neglecting differences in the implications of academic failure between the WEIRD societies and the CHCs, that might lead to overgeneralization of learner characteristics across cultural contexts. This overgeneralization also happened in the 2018 Programme for International Student Assessment (PISA; OECD, 2019). The 2018 results seemed to concur with the self-worth theory that students in high-performing Asian countries, such as Singapore, Korea, Japan, and Taiwan, shared similar characteristics of the overstrivers of the quadripolar model. These Asian students scored above the OECD average, and so did their fear of failure; in addition, these students were less satisfied with their lives (OECD, 2019). The 2018 PISA results might attempt to portray the well-performing Asian students as overstrivers, who were caught in the conflict between FF and HS and were vulnerable to emotional distress (Covington, 1992).

In summary, according to the self-worth theory, FF would lead to an avoidance tendency, thus preventing students from taking academic risks (Clifford, 1988, 1991). Failure-fearing students were generally vulnerable to emotional distress and learned helplessness (Covington and Omelich, 1991; Martin and Marsh, 2003; De Castella et al., 2013). In addition, students who were high in FF but low in HS seemed to be at greatest risk both academically and emotionally (Covington and Omelich, 1991; De Castella et al., 2013). Success-oriented students would be more likely to approach academic challenges, feel capable of controlling their academic performance, and be in better mental health (Covington, 1992).

### 1.2. Learners’ perceptions of academic failure in the CHCs: Role obligations and feelings of indebtedness

Heine et al. (2001) have indicated that the perceived consequences of academic failure differed across cultural contexts. The self-worth theory regards academic performance as ability estimates and interprets academic failure as a threat to personal worth because it implies low ability and will elicit shame and humiliation (Covington and Omelich, 1979). However, researchers have argued that this ability-based interpretation might not be fully applicable to the CHC contexts, where relationalism and role obligations have been an integral part of the cultural and social values (Hwang, 2000; Heine et al., 2001; Fwu et al., 2021). According to the role obligation theory of self-cultivation (ROT; Fwu et al., 2018, 2021), the CHC learners tend to perceive academic achievement and failure from personal and interpersonal perspectives. The former refers to personal obligations to exert oneself toward the ultimate good, and the latter refers to fulfilling filial obligations to parents by achieving academic excellence (Fwu et al., 2017, 2018). The two aspects will be explained in detail below.

Individuals are ascribed a role to play within hierarchical social relationships and are expected to fulfill their role obligations to achieve personal and interpersonal harmony (Heine et al., 2001; Hwang, 2001). One of the primary role obligations of the CHC learners is to study hard and strive for academic excellence (Fwu et al., 2021). Chen et al. (2009) found that the CHC learners tended to see academic achievement as a vertical goal that carries strong social and especially parental expectations (Chen et al., 2009). The role obligation of pursuing academic excellence is further reinforced by socialization processes in school, thus becoming a consensual goal shared by the CHC learners and their parents (Chen et al., 2009). In other words, the social and parental expectations will gradually develop into a perceived role obligation of CHC learners, making them not only feel expected but also obligated to work hard to excel academically (Heine et al., 2001; Chen et al., 2009). Fwu et al. (2021) have applied the ROT and provided evidence supporting that CHC learners tend to persist in the face of academic failure rather than give up.

ROT also has a personal aspect that involves self-exertion and self-cultivation (Fwu et al., 2017). These personal obligations can be traced back to ancient Chinese axioms, such as “cultivate oneself toward the ultimate good” (止於至善) and “continuously improve yourself each and every day” (茍日新, 日日新, 又日新; Fwu et al., 2021, p. 5). The role obligation of achieving academic excellence requires the CHC learners to exert and improve themselves constantly throughout schooling and even in higher education. The constant self-exertion toward academic excellence is a means through which the CHC learners fulfill the filial obligations to their parents. Given that achieving academic excellence has become a consensually shared goal between the learners and their parents, the learners will also expect themselves to work harder and perform better.

Given that the pursuit of academic excellence is perceived as role obligations, academic failure will trigger self-reflection (e.g., reflect on yourself when you failed行有不得反求諸己) and induce two types of negative emotions, including indebtedness to self (愧對自己) and indebtedness to parents (愧對父母; Fwu et al., 2018). The feelings of indebtedness are rooted in the Confucian relationalism and intertwined with child–parent role obligations (Chao, 1996; Bedford and Hwang, 2003; Kang and Larson, 2014; Kang and Raffaelli, 2015). Indebtedness is different from guilt in that guilt is triggered by wrongdoings and entails “the feeling of responsibility for transgression” (Bedford and Hwang, 2003, p. 128), whereas indebtedness is induced by failure to fulfill role obligations in social relationships. Fwu et al. (2021) has explained the feelings of indebtedness from personal and interpersonally perspectives. Interpersonally, indebtedness to parents is associated with failure to be a filial child to repay parents for their sacrifice for the family and to make them proud (Kang and Larson, 2014; Kang and Raffaelli, 2015; Fwu et al., 2021). Personally, indebtedness to self is due to failure to exert and cultivate oneself toward the ultimate good (Fwu et al., 2021). Unlike the self-worth theory that sees academic failure as a threat to personal worth, academic failure will have moral implications in the CHCs because it represents failure to fulfill role obligations of being a filial child and a better self (Chao, 1996; Kang and Larson, 2014; Fwu et al., 2021).

In their studies of the relationship between academic failure and feelings of indebtedness, Fwu et al. (2018) found that when academic failure was attributed to lack of effort, it would trigger indebtedness to parents and indebtedness to self; however, only the latter would activate subsequent effort-making, indicating that “the self is the agent for actions” (Fwu et al., 2018, p. 28). Fwu et al. (2021) further substantiated the significant effect of indebtedness to self on effort-making after academic failure. These results have shown that although indebtedness to self is a negative emotion in nature, it would also motivate an individual to exert and improve subsequently rather than give up.

### 1.3. The present study

A review of the literature has shown that the quadripolar model of achievement motivation was built upon the self-worth theory, and the four categories of the model have been applied to examine learner characteristics mainly in the WEIRD contexts. Even in cross-cultural studies (De Castella et al., 2013; OECD, 2019), there seemed to be no cultural differences in categorization and in learner characteristics. Given that the relationalism and role obligations have been an integral part of the CHCs, there are fundamental differences in learners’ perceptions of academic failure between the CHCs and the WEIRD contexts as explained by the ROT and the self-worth theory, respectively. Using the self-worth theory alone might lose the culturally unique values, such as feelings of indebtedness to self and parents and might overlook the characteristics of the CHC learners. It is necessary to re-examine the applicability of the quadripolar model to better understand the dynamics of FF and HS and to explore how the dynamics contribute to various learner characteristics in the CHCs.

Firstly, we hypothesized that the CHC learners would perceive academic failure from both personal and interpersonal perspectives as predicted by the ROT. Thus, FF would have two sub-factors. This would be different from the studies built upon the self-worth theory that regards academic failure as a general personal factor. Secondly, given the fundamental differences in the perceptions of academic failure, this study also hypothesized that there might be additional categories emerging from the CHC sample, apart from the four existing categories, i.e., the overstrivers, self-protectors, failure acceptors, and optimists. Thirdly, we hypothesized that the additional categories might constitute a significant part of the CHC sample according to Chen et al. (2021). It was found that 43% of the CHC students in their sample held the role obligation of self-cultivation. These students showed a stronger approach tendency than avoidance toward difficult situations and had less emotional distress than those who had a fixed mindset of ability (Chen et al., 2021). Fourthly, categories emerging from the CHC sample of the current study would demonstrate varying learner characteristics as measured by (1) risk-taking tendency, (2) learned helplessness, and (3) emotional distress. These characteristics would partly concur with those that have been identified in the previous studies (Covington and Omelich, 1991; De Castella et al., 2013). Additional categories, if any, emerging from the current study would demonstrate unique learner characteristics because of the differences in interpreting academic failure between the self-worth theory and the ROT.

## 2. Materials and methods

### 2.1. Participants and procedure

Participants of the study were undergraduate seniors, i.e., students who were in their fourth year of study in a university. Data collection was undertaken in a public research-intensive university in northern Taiwan (hereafter the University). The University has been characterized by its academically challenging and competitive environment for students, making it a suitable site for the study. We acknowledged that failure experiences might be common among students across academic levels. We chose undergraduate seniors for two reasons. First, these students had accumulated more failure/success learning experiences than did their junior peers. In addition, academic risk-taking tendency was one of the criterion variables we measured. Given that challenging academic tasks, such as capstone courses, projects, or undergraduate research were an important part of studies in the senior year at the University, the undergraduate seniors would be ideal participants for the study.

An online invitation was sent to 4,364 eligible undergraduate seniors *via* the University’s email system, with 1,311 students responding to the survey. The overall response rate was 30.04%. A total of 64 participants failed the attention check item and were excluded, resulting in a valid sample of 1,247 undergraduate seniors. Participation in the survey study was entirely voluntary. Prior to answering the questionnaire, all participants gave an informed consent to participate in the study. The participants completed the questionnaire online and were allowed to submit it once. By completing the questionnaire, they could decide whether to participate in a lucky draw to win a token of appreciation. The study was granted ethical approval (NTU-REC no. 202111HS007) by the Research Ethics Committee at the National Taiwan University.

### 2.2. Measures

The questionnaire consisted of five measures, including (1) fear of failure, (2) hope of success, (3) academic risk-taking tendency, (4) learned helplessness, and (5) emotional distress as explained in detail below. The first four measures used a 6-point Likert scale, with 1 representing strongly disagree and 6 representing strongly agree. Emotional distress was measured by the Brief Symptom Rating Scale that used a 5-point Likert scale, with 0 representing none and 4 representing very severe (Lee, 2018). The measures were translated from English to Chinese (measures 1, 2, 3, and 4) and Chinese to English (measure 5) by the first author. The translated items were backward translated by two research assistants who were fluent in English and Chinese and then checked by the first author.

**Fear of failure (FF)** was measured by nine items. Five of them came from the short-form of the Performance Failure Appraisal Inventory (PFAI-S) developed by Conroy et al. (2002). These items were used in De Castella et al. (2013) to measure FF. The five items corresponded to five negative consequences of failure: (a) experiencing shame and embarrassment, (b) devaluing one’s self-estimate, (c) having an uncertain future, (d) losing social influence, and (e) upsetting important others (Conroy et al., 2002, p. 77). Sample items include “When I am failing, I worry about what others think about me” and “When I am failing, I am afraid that I might not have enough talent” (Conroy et al., 2002, p. 90). Considering the feelings of indebtedness are significant negative emotions that the CHC learners would experience after academic failure, we added four items, with two of them assessing indebtedness to parents (e.g., When I am failing, I feel indebted to my parents.) and with the other two items assessing indebtedness to self (e.g., When I am failing, I feel indebted to myself.), in the face of failure (α = 0.88).

**Hope of success (HS)** was measured by the Hope Scale developed by Snyder et al. (1991). Eight items were adopted to assess an individual’s belief about achieving goals through their agency and perceived pathways of achieving the goals. Sample items include “I can think of many ways to get out of a jam” (pathways), and “I energetically pursue my goals” (agency; α = 0.89).

**Academic risk-taking tendency** was measured by the School Failure Tolerance (SFT) scale developed by Clifford (1988). The SFT has three subscales, including failure feelings, preferred difficulty, and actions in response to academic challenges. It has been found to be effective in predicting academic risk-taking, with the subscale of preferred difficulty demonstrating better effects (Clifford et al., 1988, 1989; Tan et al., 2017). The current study focused academic risk-taking on students’ choices between challenging and easier tasks and on their choices of action between persistence or quitting. Thus, we excluded the failure feelings subscale. Twelve items were adopted from the preferred difficulty subscale (e.g., “I like to try difficult assignments even if I get some wrong”) and the action subscale (e.g., “When I make mistakes in a difficult task, I just keep trying and trying”; α = 0.88).

**Learned helplessness** was measured by the Master-orientation subscale developed by (Nurmi et al., 1995). The subscale was part of the Strategy and Attribution Questionnaire (SAQ) that assessed an individual’s perceived control over their academic studies and outcomes. Nurmi et al. (1995) defined those who responded having little or no control as learned helplessness (p. 111). Seven items were used to measure learned helplessness. Sample items include “I do not have the means to affect the way my studies go” and “Careful preparation for an exam leads to good results” (α = 0.78).

**Emotional distress** was measured by the Brief Symptom Rating Scale (BSRS-5; Lee, 2018). The BSRS-5 had five items and have been used widely to assess levels of emotional distress (Chen et al., 2005, 2009). Participants responded the five items on a scale of zero to four with zero representing none and four very severe. The BSRS-5 (Lee, 2018) used a four-level scale to represent mental health based on the total score of the five items. According to Lee (2018), a total score of zero to five points represented a good status of mental health (Level 1). As the level went up, a need of psychological consultation and clinical interventions increased accordingly. Six to nine points showed mild emotional distress (Level 2). A total of 10 to 14 points indicated mid-level emotional distress that required psychological or clinical consultation (Level 3). A total of 15 points and above indicated severe emotional distress and very high need of clinical interventions (Level 4). Sample items include “Get stressed out or lose temper easily” and “Feel inferior to others” (α = 0.89).

### 2.3. Statistical analysis

Descriptive statistics of the five variables were shown in Appendix 1. A correlation matrix was shown in Appendix 2. Confirmatory factor analysis (CFA) and latent class analysis (LCA) were conducted to examine our hypotheses. We performed CFA to examine whether FF has two sub-factors as predicted by the ROT, including a personal and an interpersonal factor. LCA was then performed to testify whether the original quadripolar model was applicable to the CHC sample. LCA has been used to examine “the underlying subgroups” of a population under investigation (Jeon et al., 2017, p. 912). In the present study, we used LCA to explore unobserved learner categories (i.e., latent classes) and to classify participants based on their responses to FF and HS (Jeon et al., 2017). The present study was built on the hypothesis that the CHC learners would perceive academic failure from two perspectives, personally and interpersonally. However, academic failure was mostly treated as a general factor in the previous studies guided by the self-worth theory (Conroy et al., 2003; De Castella et al., 2013; Balkis and Duru, 2019). Thus, we randomly split the sample into two subsamples (hereafter the subsample one and subsample two) to examine whether the quadripolar model (Covington and Omelich, 1991) could be replicated and to verify our hypotheses as guided by the ROT. All analyses were performed using SAS 9.4 (Lanza et al., 2007).

## 3. Results

### 3.1. A preliminary analysis of the subsample one

The subsample one consisted of around 30% of the participants (*n* = 382). This preliminary analysis was aimed at examining whether the Covington’s quadripolar model (Covington and Omelich, 1991) could be replicated in the present study. We adopted the original short-form of the Performance Failure Appraisal Inventory (PFAI-S; Conroy et al., 2002), and excluded the four items of indebtedness to self and significant others.

Results of the CFA showed that a two-factor model of FF fitted the data better than did a one-factor model. The two subfactors corresponded to a personal and an interpersonal aspect, respectively. The fitness of the two-factor model was acceptable, *χ*^2^ = 24.73, df = 4, *p* < 0.0001, comparative fit index (CFI) = 0.97, root mean square error of approximation (RMSEA) = 0.10, standardized root mean squared residual (SRMR) = 0.03 (Browne and Cudeck, 1993; Hu and Bentler, 1999; Fan and Sivo, 2007; Hooper et al., 2008). The one-factor model yielded poorer model fit, *χ*^2^ = 44.58, df = 5, *p* < 0.0001, CFI = 0.95, RMSEA = 0.13, SRMR = 0.17. The relatively high RMSEA was caused by the small degree of freedom (Chen et al., 2008; Kenny et al., 2015). The chi-square difference test also indicated that two-factor model was significantly better than the one-factor model (*χ*^2^ = 19.84, df = 1, *p* < 0.0001). In summary, the CFA results showed that the CHC learners would perceive FF from the personal and interpersonal perspectives as the ROT predicted.

LCA was then performed to classify participants based on patterns of responses to FF and HS. In this preliminary analysis of the subsample one, we examined whether the original quadripolar model could be replicated. Prior to LCA, we transformed the 6-point scales of FF and HS into 2-point scales using a cut-off score of 3.5. Scores equal to or higher than 3.5 were coded as 1. Scores lower than 3.5 were coded as 0. The cut-off score was set at 3.5 because we used a 6-point Likert scale, with scores of 3 (i.e., somewhat disagree) and below representing negative responses and scores of 4 (i.e., somewhat agree) and above indicating positive responses.

The LCA results indicated that in order to replicate the four learner categories as did in Covington and Omelich (1991), we needed to adopt a six-class model rather than a four-class one. In addition to the four categories in the quadripolar model, the six-class model also included two additional categories. In summary, the CFA and LCA results supported our hypotheses. The CHC learners would perceive academic failure personally and interpersonally rather than regarding it as a general personal factor. The LCA results also indicated that the quadripolar model might not suffice to represent the CHC sample. There existed two additional learner categories that might be unique to the CHC sample.

### 3.2. The main study

The main study used the subsample two which consisted of around 70% of the participants (*n* = 865). The main study aimed to examine whether the two-factor model of FF fitted the data better and whether the six-class model held as suggested by the preliminary analysis. We used the adapted measure of FF, that included the original five items of the PFAI-S and the four items of indebtedness to self and significant others.

Concurring with the results reported in the section 3.1, the CFA results once again suggested a two-factor model of FF. For both the one-factor and two-factor CFA models, we specified a covariance parameter between the residuals of the items fear 2 and fear 8 because the two items were highly similar to each other in their contents. The two-factor model consisted of a personal and an interpersonal subfactor. The fitness of the two-factor model was acceptable, *χ*^2^ = 324.50, df = 25, *p* < 0.0001, CFI = 0.94, RMSEA = 0.10, SRMR = 0.04. The one-factor model yielded poorer model fit, *χ*^2^ = 387.05, df = 26, *p* < 0.0001, CFI = 0.93, RMSEA = 0.11, SRMR = 0.17. The chi-square difference test also indicated that two-factor model was significantly better than the one-factor model (*χ*^2^ = 62.55, df = 1, *p* < 0.0001). The CFA results supported our hypothesis that the CHC learners perceive FF from the personal and interpersonal perspectives as predicted by the ROT.

#### 3.2.1. Latent class analysis

The data were fitted to five models with the number of classes ranging from five to nine. The model fit indices of each model were shown in Table 1. The five models were further evaluated against the original quadripolar model (Covington and Omelich, 1991) and the ROT (Fwu et al., 2021). Eventually, the six-class model was determined to be the most fit and parsimonious. The LCA results were supported by the theories and consistent with the results reported in the section 3.1.

**Table 1:** Model fit indices and degree of freedom of the five models.

# class	5	6	7	8	9
Log-likelihood	−9330.04	−9269.52	−9210.2	−9154.87	−9112.21
BIC	4993.55	4999.84	5008.54	5025.22	5067.21
CAIC	5082.55	5106.84	5133.54	5168.22	5228.21
Entropy	0.82	0.82	0.82	0.82	0.83
*df*	130982	130964	130946	130928	130910

As shown in Table 2, the six-class model included the four existing categories of the quadripolar model, i.e., the overstrivers, self-protectors, failure acceptors, and optimists. Two additional categories emerged and were labelled as the CHC overstrivers and the CHC self-protectors, respectively. It is worth noting that the quadripolar model only constituted 50.3% of the subsample two. The two CHC categories altogether accounted for the remaining 49.7%. The two CHC categories constituted a significant part of the CHC sample. Among the six categories, the CHC overstrivers constituted the highest proportion (29.9%), followed by the overstrivers (22.7%), the CHC self-protectors (19.8%), the self-protectors (12.5%), the optimists (11.6%), and the failure acceptors (3.5%).

**Table 2:** Conditional probabilities and group proportions of the six observed latent groups.

				Over-striver	Self-protector	Optimist	Failure acceptor	CHC over-striver	CHC self-protector
Factors		Items	Proportion	22.7%	12.5%	11.6%	3.5%	29.9%	19.8%
Fear of failure	Failing oneself	When I am failing, I worry about what others think about me.	fear1	**0.995**	**1.000**	0.289	0.603	**0.883**	**0.863**
		When I am failing, I am afraid that I might not have enough talent.	fear3	**0.983**	**1.000**	0.480	**0.722**	**0.944**	**0.896**
		When I am failing, I am afraid that I might not do my best.	fear4	**0.900**	**0.882**	0.265	0.553	**0.746**	**0.730**
		When I am failing, I feel indebted to myself.	fear9	**0.979**	**0.971**	0.240	0.299	**0.772**	**0.734**
	Failing significant others	When I am failing, it upsets my plan for the future.	fear5	**0.880**	**0.946**	0.135	0.441	0.559	0.487
		When I am not succeeding, people are less interested in me.	fear6	**0.881**	**0.855**	0.005	0.175	0.409	0.484
		When I am failing, important others are disappointed.	fear7	**0.963**	**0.923**	0.002	0.108	0.429	0.404
		When I am failing, I worry that it will embarrass my family.	fear2	**0.944**	**0.825**	0.055	0.174	0.268	0.226
		When I am failing, I feel indebted to my parents.	fear8	**0.927**	**0.902**	0.030	0.094	0.205	0.190
Hope of success	Pathway	I can think of many ways to get out of a jam.	pathway1	**0.996**	0.565	**0.999**	0.142	**1.000**	**0.886**	
		There are lots of ways around any problem.	pathway2	**0.966**	0.425	**0.970**	0.219	**0.963**	**0.764**	
		I can think of many ways to get the things in life that are most important to me.	pathway3	**0.993**	0.382	**0.999**	0.050	**0.993**	0.668	
		Even when others get discouraged, I know I can find a way to solve the problem.	pathway4	**0.960**	0.320	**0.983**	0.156	**0.939**	0.671	
	Agency	I energetically pursue my goals.	agency1	**0.979**	0.457	**0.956**	0.215	**0.977**	0.595		
		My past experiences have prepared me well for my future.	agency2	**0.832**	0.243	**0.919**	0.167	**0.903**	0.392		
		I meet the goals that I set for myself.	agency4	**0.814**	0.286	**0.901**	0.086	**0.880**	0.437		
		I’ve been pretty successful in life.	agency3	0.451	0.099	0.558	0.151	0.408	0.158

Conditional probabilities greater than 0.700 were marked in bold.

The major differences between the two CHC categories and the four categories of the quadripolar model lied in their responses to FF. The two subfactors of FF were labelled as failing oneself and failing significant others, respectively. The two items of indebtedness to self were assigned to the subfactor of failing oneself. The other two items of indebtedness to significant others were assigned to the subfactor of failing significant others. The overstrivers and self-protectors had high responses to both failing oneself and failing significant other, whereas the CHC overstrivers and CHC self-protectors responded high to failing oneself only.

#### 3.2.2. Profile analysis and the criterion variables

**Figure 2:**
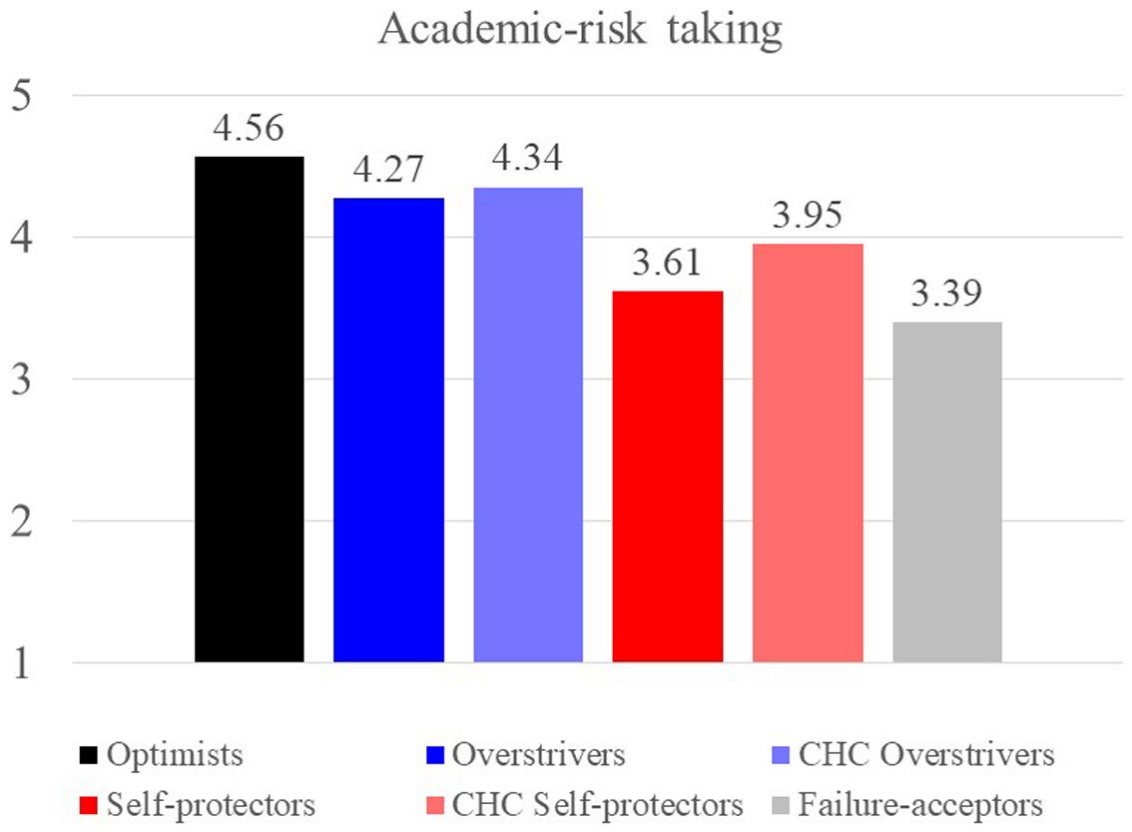
Academic-risk taking tendency across categories.

Profile analysis was performed to examine whether the six categories had distinctive characteristics as measured by academic risk-taking tendency, learned helplessness, and emotional distress. As shown in Figure 2, academic risk-taking was associated with HS. Students who were high in HS, i.e., the optimists, CHC overstrivers, and overstrivers, demonstrated stronger approach tendencies toward academic risk-taking than their counterparts who were low in HS, i.e., the CHC self-protectors, self-protectors, and failure-acceptors. The profile analysis also showed consistent results with the previous studies indicating that the self-protectors and overstrivers were at greater risk of learned helpless (De Castella et al., 2013) and emotional distress (Covington and Omelich, 1991). The CHC overstrivers and the CHC self-protectors demonstrated lower levels of learned helpless and emotional distress compared with the corresponding categories in the quadripolar model (i.e., the overstrivers and the self-protectors respectively). The optimists were the least helpless and emotionally distressed category as expected. The detailed results of the three criterion variables were explained below.

**Academic risk-taking.** As shown in Figure 2, it was the HS that led student to an approach tendency toward academic risks. Among the three categories that were high in HS, the optimists showed the strongest risk-taking tendency, followed by the CHC overstrivers and the overstrivers. The other three categories that were low in HS, i.e., the CHC self-protectors, the self-protectors, and the failure acceptors, were less likely to approach academic risks, with the failure acceptors showing the least risk-taking tendency.

**Figure 3:**
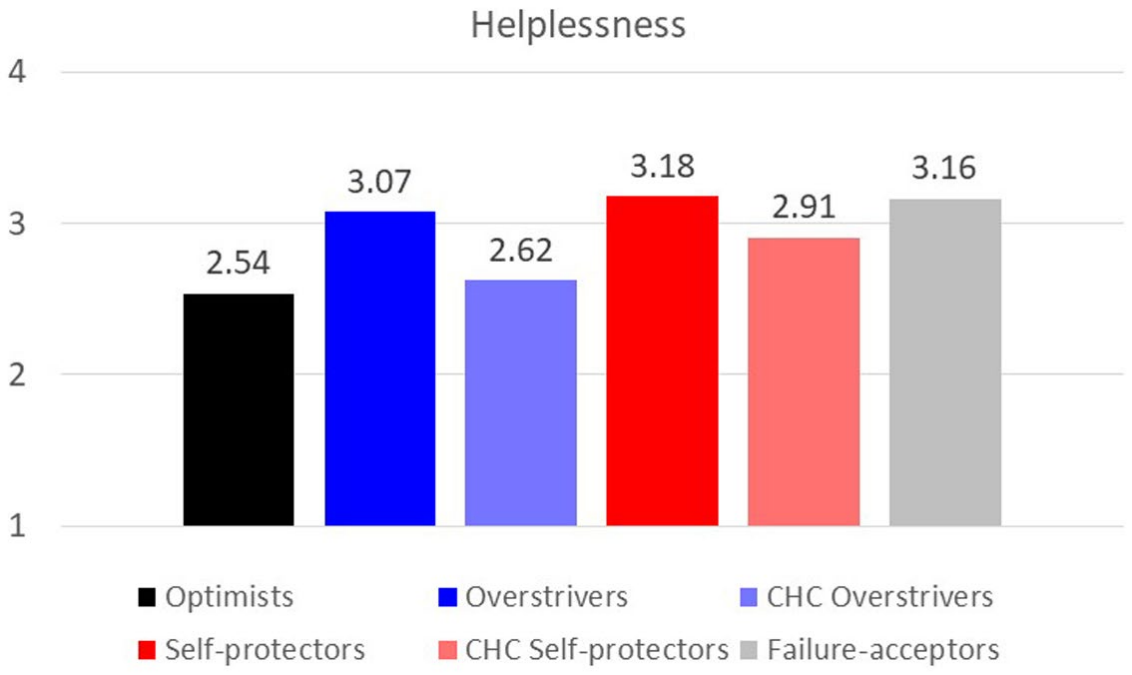
Levels of learned helplessness across categories.

**Learned helplessness.** Figure 3 showed the varying levels of learned helplessness across the six categories. The results were consistent with the previous studies showing that the self-protectors were at greatest risk of learned helpless (De Castella et al., 2013), followed by the failure acceptors, the overstrivers, and the CHC self-protector. It is worth noting that the CHC overstrivers and the optimists demonstrated similar levels of learned helplessness.

**Figure 4:**
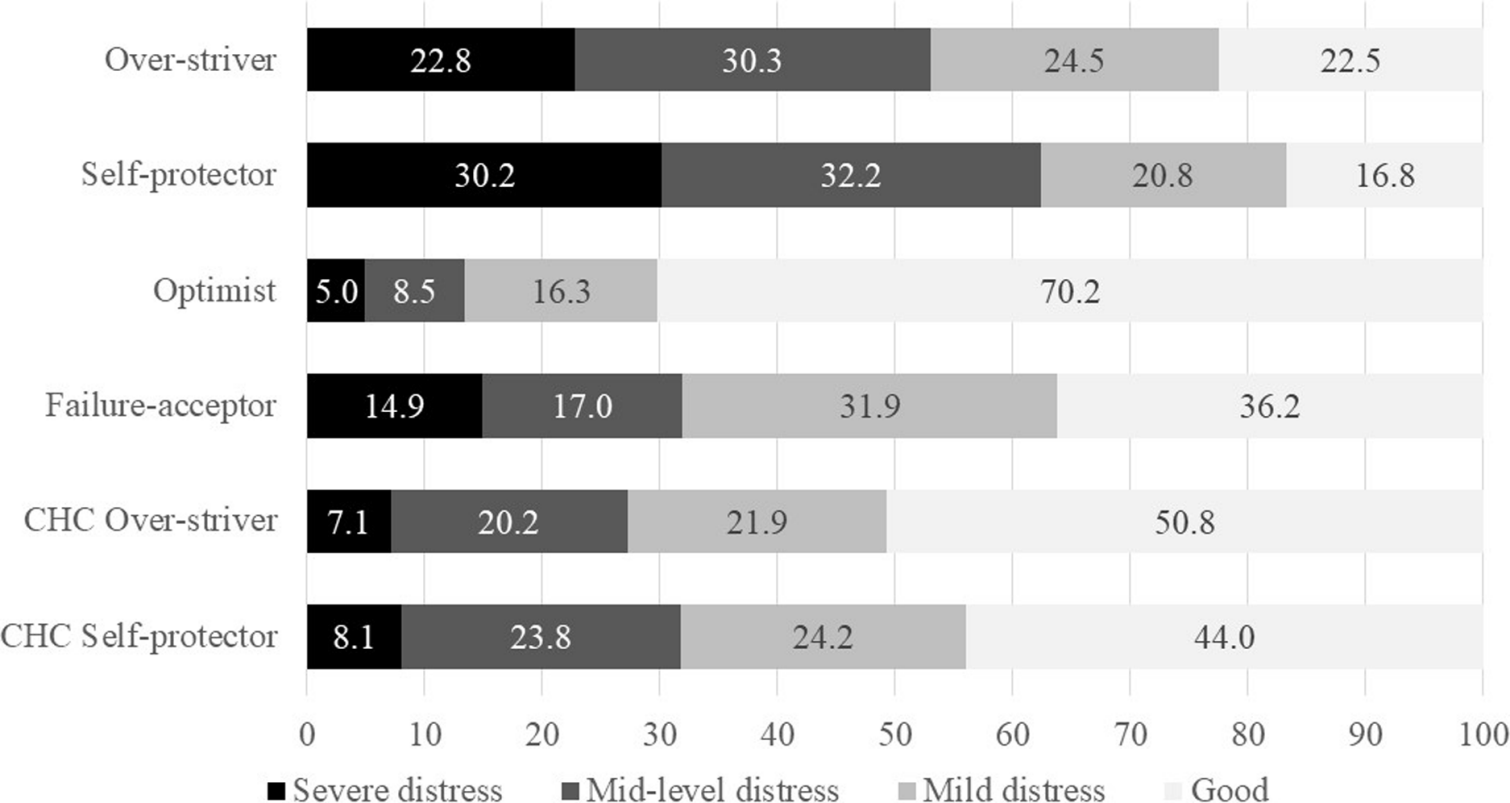
Levels of emotional distress across categories (by percentage).

**Emotional distress.** Figure 4 displayed the proportions of emotional distress levels across the categories. The results substantiated the association between FF and emotional distress. The self-protectors and overstrivers were high in FF and were emotionally distressed in general. Over 62% of the self-protectors and over 53% of the overstrivers were in mid-level to severe emotional distress.

The two CHC categories were similar in distributions across the four distress levels. In addition, compared with their corresponding categories in the quadripolar model, they were less emotionally distressed. Around 27% of the CHC overstrivers and 32% of the CHC self-protectors were in mid-level to severe emotional distress. The proportions of the first level were also noteworthy. Around 51% of the CHC overstrivers and 44% of the CHC self-protectors were in good mental health, whereas around 22% of the overstrivers and 17% of the self-protectors were. Optimists were in general in good mental health as expected.

## 4. Discussion

Results of the preliminary analysis and the main study supported the four hypotheses. First, the two-factor model of FF indicated that CHC learners perceive academic failure from both personal and interpersonal perspectives as suggested by the ROT. Second, LCA results suggested a six-class model that consisted of the four categories in the quadripolar model and two additional CHC categories. Third, the two additional CHC categories constituted 49.7% of the sample, showing that the original quadripolar might not fully representative of the CHC sample. Fourth, the six categories demonstrated varying learner characteristics as measured by the three criterion variables. The characteristics of the four quadripolar categories were consistent with those found in the previous studies (Covington and Omelich, 1991; De Castella et al., 2013). The two additional CHC categories revealed unique characteristics that could differentiate them from their corresponding categories in the quadripolar model. Theoretical significance of the study and practical implications derived are discussed as follows.

### 4.1. FF as a general factor vs. with two subfactors

As shown in sections 3.1 and 3.2, the CFA results consistently supported our first hypothesis that FF included two subfactors as predicted by the ROT and that the CHC learners would perceive academic failure from both personal and interpersonal perspectives (Fwu et al., 2018, 2021). Furthermore, the two types of negative emotions, i.e., indebtedness to self and to parents were assigned to the subfactors of failing oneself and failing significant others, respectively. Given the fundamental differences in the structure of FF, the LCA results also supported our hypothesis that there existed additional categories in addition to the four categories in the quadripolar model. The two CHC categories, including the CHC overstrivers and the CHC self-protectors, emerged from the two subsamples consistently. The four quadripolar categories only accounted for half of the sample (50.3%), and the two additional CHC categories constituted the other half (49.7%). These results pointed out the inadequacy of the self-worth theory in explaining achievement motivation in the CHCs where relationalism and role obligations constitute an integral part of the cultural and social values (Hwang, 2000; Heine et al., 2001; Fwu et al., 2021).

Low endorsement of the subfactor of failing significant others differentiated the two additional CHC categories from their corresponding quadripolar categories. The CHC overstrivers and the CHC self-protectors only responded high to the subfactor of failing oneself, whereas the overstrivers and self-protectors responded high to both subfactors. The LCA results suggested that the two CHC categories had a stronger tendency toward self-reflection (Fwu et al., 2018) than toward social obligations after academic failure.

A possible explanation of the low endorsement of failing significant others as found in the two additional CHC categories might be that the participants of the present study were undergraduate seniors who were about to complete their undergraduate studies. University education could be challenging particularly in a research-intensive university where the current study was undertaken. It seemed reasonable to assume that the students who had high endorsement of failing oneself but low of failing significant others were able to find personal meanings in their undergraduate studies, that could drive them to constantly exert and improve themselves to sail through academic challenges and failure. On the other hand, the obligation to fulfill expectations of significant others were secondary.

However, it was worth noting that the two quadripolar categories, i.e., the overstrivers and self-protectors demonstrated high endorsement of the two subfactors of FF in the present study, and that the two categories constituted around 35% of the sample. It seemed that not only fear of failing oneself but also fear of failing significant others was a significant characteristic of these students.

### 4.2. Differences in learner characteristics between the two CHC categories and the two corresponding quadripolar categories

Results of the profile analysis in the main study were consistent with the previous studies that FF would lead to an avoidance tendency toward academic risk-taking (Clifford, 1988, 1991; Tan et al., 2017). However, the results of the profile analysis also revealed differences between the two CHC categories and their corresponding quadripolar categories. The major differences were their levels of emotional distress. The previous studies have shown that the overstrivers and self-protectors were vulnerable to anxiety due to their high FF (Covington and Omelich, 1991; Covington, 1992; Martin and Marsh, 2003). The present study found that although the overstrivers and the self-protectors were emotionally distressed in general, the CHC overstrivers and CHC self-protectors were not. Around half of the CHC overstrivers and around 44% of the CHC self-protectors showed good mental health. The results suggest that fears of failing students themselves might not necessarily result in poorer mental health as did FF in general (Covington and Omelich, 1991; Covington, 1992). This might debunk the myth that Asian students would strive for academic excellence at the cost of their psychological wellbeing as implied in the PISA 2018 report (OECD, 2019).

In addition to levels of emotional distress, the present study also found difference in academic risk-taking tendency between the self-protectors and the CHC self-protectors. The two categories were both high in FF and low in HS, and according to the self-worth theory, these students would demonstrate a strong avoidance tendency toward academic risk-taking. However, the profile analysis showed that the CHC self-protectors demonstrated higher academic risk-taking tendency than did the self-protectors. The results seem to concur with the previous studies that indebtedness to self could trigger subsequent effort making after academic failure, whereas indebtedness to parents could not (Fwu et al., 2018, 2021). Given that the CHC self-protectors only responded high to the subfactor of failing oneself, it is reasonable to assume that the intention to avoid failing oneself might not necessarily lead to an avoidance tendency toward academic challenges. Instead, it might help facilitate academic risk-taking to a lesser extent compared with HS.

### 4.3. Practical implications

The findings of the present study would offer several practical implications. Students are usually encouraged to strive for and celebrate academic excellence; however academic risk-taking and failure are inevitable in pursuit of higher education. Given that around 85% of the CHC students in the main study were high in FF, including the two overstrivers and two self-protectors, it is necessary for higher education institutions to provide students with ample psychological support and mental health resources. It would be helpful to create failure-friendly environments where students feel free to share and discuss failure experiences with peers and teachers. Learning environments that encourage an open discussion on failure experiences would remind students that failure happens and is normal and help them see failure in a constructive manner to channel their fears into opportunities.

In addition, in order to enhance HS of students, Covington and colleagues (Covington, 1992; Covington et al., 2017) have advocated engaging students with inquiry-based activities, such as capstone tasks. Inquiry-based activities were grounded in the belief that learning takes place through discovery rather than transmission. These activities emphasize not only learning products but also processes. They allow students to choose topics of inquiry to tap into curiosity and intrinsic motivation. An exploration of the effects of this pedagogical approach would be helpful in finding a way to encourage students to approach learning with hope of success.

Lastly, we would like to argue that although the failure acceptors constituted a very small part of the sample in the main study (3.5%), these students required more attention than they had received in the literature. The failure acceptors showed the least risk-taking tendency among the six categories, that could disadvantage them in such competitive learning environments as research-intensive universities where academic challenges were inevitable (Tan et al., 2017). Engaging the failure-accepting students with inquiry-based learning activities might help them find an area of interest as sources for intellectual and emotional satisfaction.

### 4.4. Limitations and future research

This study has several limitations. First, the data were collected from one research-intensive university. Generalization of the findings should be treated with caution considering the diversity of demographics and variations in learner characteristics across research-intensive universities in the CHCs and other cultural settings. Secondly, although the LCA results indicated that the CHC overstrivers and CHC self-protectors were two different categories and that their academic risk-taking tendency differed, their perceived learned helplessness and levels of emotional distress were similar. Future studies might explore learner characteristics that could further differentiate between the two CHC categories. Thirdly, this study found that students with different achievement motivation demonstrated different levels of emotional distress and learned helpless and responded to academic risk-taking differently. It remains unclear where this mechanism might be altered through pedagogical interventions. Future studies might explore whether the proposal of inquiry-based learning by Covington and colleagues (Covington, 1992; Covington et al., 2017) stands to provide illuminating implications for teaching and learning in higher education.

## 5. Conclusion

This study examined the applicability of the quadripolar model of achievement motivation (Covington and Omelich, 1991) in a CHC higher education institution characterized by its academically challenging and competitive environment for the students. Apart from the four categories of the quadripolar model, two CHC categories emerged from the sample. The two CHC categories constituted half of the sample and demonstrated different learner characteristics compared with their corresponding quadripolar categories. The results may help debunk the myth that learner characteristics in the CHCs are identical to those observed in the WEIRD contexts. The fundamental differences in FF further indicated the inadequacy of the self-worth theory in explaining achievement motivation in the CHCs where relationalism and role obligations are significant parts of the cultural traditions. The study demonstrates the importance of considering cultural differences rather than entirely transplanting models built primarily upon the WEIRD contexts to non-WEIRD settings.

## Data availability statement

The original contributions presented in the study are included in the article/supplementary material, further inquiries can be directed to the corresponding author.

## Ethics statement

The studies involving human participants were reviewed and approved by Research Ethics Committee, National Taiwan University (NTU-REC no. 202111HS007). The patients/participants provided informed consent in electronic format to participate in this study.

## Author contributions

GL and B-JF conceptualized the study. GL, B-JF, and T-RY formulated the hypotheses and designed the study, with Y-KC contributing to the discussions. T-RY and Y-KC organized and performed the statistical analyses. GL, B-JF, and T-RY interpreted the results and findings, with Y-KC contributing to the discussions. Drafts of the manuscript were written by GL and revised by B-JF. All authors contributed to the article and approved the submitted version.

## Funding

This study was sponsored by the Center for Teaching and Learning Development, National Taiwan University and was supported by the research grant awarded to the corresponding author B-JF, under award No. MOST 110-2410-H-002-080-SS3 by the National Science and Technology Council in Taiwan. The funders had no role in the design of the study, analyses and interpretation of the data, preparation of the manuscript or decision to publish the manuscript.

## Conflict of interest

The authors declare that the research was conducted in the absence of any commercial or financial relationships that could be construed as a potential conflict of interest.

## Publisher’s note

All claims expressed in this article are solely those of the authors and do not necessarily represent those of their affiliated organizations, or those of the publisher, the editors and the reviewers. Any product that may be evaluated in this article, or claim that may be made by its manufacturer, is not guaranteed or endorsed by the publisher.

## Appendix 1

Descriptive statistics of the variables.

**Table:**

	Mean	Standard deviation	Skewness	Kurtosis	Maximum	Minimum
Fear of failure	3.91	0.95	−0.35	0.19	6	1
Hope of success	4.06	0.79	−0.42	0.84	6	1
Academic Risk taking	4.20	0.88	−0.35	0.70	6	1
Helplessness	2.86	0.96	0.41	0.18	6	1
Emotional distress	7.85	5.28	0.44	−0.71	20	0

Emotional distress was calculated by the total score of all items, and the remaining variables were calculated by the average score of all items.

## Appendix 2

Correlation matrix for the variables.

**Table:**

	FF	HS	RT	HL	ED
Fear of failure (FF)					
Hope of success (HS)	−0.21				
Academic risk taking (RT)	−0.09	0.49			
Helplessness (HL)	0.19	−0.17	−0.18		
Emotional distress (ED)	0.49	−0.24	−0.09	0.31	

All correlations significant at α = 0.01 level.
